# Analysis of Hardening Characteristics of Aged Concrete Prepared with Highly Mineralized Mine Water—A Mine in the Ordos Mining Area Is Taken as an Example

**DOI:** 10.3390/ma16062418

**Published:** 2023-03-17

**Authors:** Yunhai Cheng, Yifan Wang, Hao Shen, Junfei Zhang

**Affiliations:** 1 Mining Engineering Research Institute, Shandong University of Science and Technology, Tai’an 271001, China; 2 Coal-Mine Filling & Mining National Engineering Laboratory, Shandong University of Science and Technology, Tai’an 271001, China; 3 School of Civil and Transportation Engineering, Hebei University of Technology, Tianjin 300401, China

**Keywords:** mine water, concrete, hardening characteristics, reaction mechanism, highly mineralized

## Abstract

In order to study the hardening characteristics and formation mechanism of concrete prepared with highly mineralized mine water (which is called C_MW_ for short), four mineralized mine water mixtures with different dosages (25%, 50%, 75%, and 100%) were prepared, and concrete specimens were made using coal-based solid waste (gangue and fly ash) as the aggregate and aged for a 70 d long-age test. Strength tests, scanning electron microscopy (SEM), and X-ray diffraction (XRD) measurements were performed to determine the relationship between the hardening strength and aging time. The hardening mechanism was studied based on the changes in the characteristic composition and microstructure. The results showed that, compared with the two-stage hardening in σ_C_ seen in conventional concrete prepared with ground purified water, drinking water, or surface water (which is called C_N-MW_ for short), σ_C_ in our experiments had three-stages. The stages included a growth period (0~28 d), in which σ_C_ of the 28 d concrete samples prepared with mine water dosages of 25%, 50%, 75%, and 100% increased by 18.0%, 36.4%, 57.2%, and 72.7%, respectively, compared with that of C_N-MW_; a rapid decline period (28~56 d), in which σ_C_ at 56 d decreased by 47.7%, 43.2%, 36.0%, and 30.5%, respectively, and finally, the stable period (56~70 d~long-age), in which the strength σ_C_ remained stable. The mechanisms of the hardening characteristics were different from those of C_N-MW_ in the three stages. In the first stage (0~28 d), Friedel’s salt and more ettringite were generated by the secondary hydration reaction, which filled the internal pores of the specimens and thus improved the compactness and σ_C_. In the second stage (28~56 d), the amount of Friedel’s salt and ettringite further increased, the crystals inside the specimens expanded, and macroscopic cracks appeared on the specimen surface, thus leading to the decrease in σ_C_. In the third stage (56~70 d~long-age), the amount of Friedel’s salt and ettringite plateaued, and σ_C_ entered a stable stage, decreasing by 52.5%, 47.8%, 40.4%, and 36.8%, respectively, compared with that of the specimens prepared without mine water. The hardening time of C_MW_ was 42 d longer than that of conventional C_N-MW_, the hardening strength decreased significantly, and the σ_C_ at the final setting time was much lower than that of C_N-MW_. Our research results provide a reference for the filling strength design of coal mine rock stratum control.

## 1. Introduction

Cemented filling in coal mines is a new green mining technology, which can not only consume a large amount of coal-based solid waste such as gangue and fly ash, protect the ecological environment of the mining area, but also effectively control the subsidence of surface mining, save filling cost, and achieve the purpose of recycling waste. Today, it has become an area of interest for researchers [1,2]. Coalmine cemented filling concrete is mainly composed of coal gangue, fly ash, cement, and mixed water [3,4]. At present, scholars’ research on filling materials mainly focuses on aggregate and cementing materials, and there are scant research into mixed water. The mixed water used in experiments is generally tap water, but in actual production, coal resources and water resources are in reverse distribution [5,6]. Faced with the shortage of water resources, higher requirements are put forward for the utilization of mine water resources. Mine water is usually characterized by high contents of suspended matter and salts, as well as being slightly acidic [7,8]. Utilization of mine water can prevent loss of water resources, avoid pollution of water environments, and alleviate the shortage of water in mining areas. Therefore, finding uses for mine water has important strategic significance for promoting the sustainable development of coal industry [9,10,11,12].

Mine water can be classified according to the characteristics of the water quality. Mine water in Eastern China can be divided into four types, namely mine water containing suspended matter, highly mineralized mine water, acidic mine water, and clean mine water [13]. For example, the mine water in the Jining No. 2 well in the Yanzhou mining area of Shandong Province has a high mineralization degree of 3133 mg/L and is classified as highly mineralized mine water. The water quality analysis of seven seepage wells in the Zibo mining area shows that the content of pyrite in coal is high, and some coal mine water in the Xinwen mining area has a high mineralization degree. According to a survey of 38 coal mines in the Shanxi, Huanglong, Shendong, and Ningdong mining areas [14,15,16], experimental research [17,18,19,20], and literature reviews [21,22], mine water in these regions has a high mineralization degree. Based on the actual properties of mine water from the Ningdong base, Bian et al. [23] put forward a development direction for treating mine water with a high mineralization degree that focuses on overcoming technical difficulties such as reasonable site selection, equipment integration, and safety guarantee. Li Li et al. [24] analyzed the utilization of mineral water in Ningxia Ningdong Coalfield, which can be divided into self-use and other uses, among which self-use is mainly used for underground grouting, dust prevention, firefighting, underground mining ground greening, and road water spraying, etc. The main uses include lake ecological water supplement and direct power supply plant after treatment, etc. Jiang Binbin et al. [25,26] proposed underground grading treatment technology and concentrated brine storage technology for high salinity mine water in Lingxin Coal Mine, which effectively realized the double-zero goal of “zero surface clean water entering the well and zero underground sewage ascending the well”. Zhao Baihang et al. [27] used coal gangue as an adsorbent to explore the removal effect and removal mechanism of dissolved organic matter in high-salinity mine water by static adsorption experiments. Liu Qi et al. [28] carried out research on hydrogeological structure characteristics and medium characteristics of high-salt mine water ectopic transfer storage reservoirs in order to ensure safe and efficient mining of coal resources and realize water reduction and emission reduction in high-salt mine wells. Gu et al. [29] proposed that underground treatment technology should be developed according to local conditions to realize large-scale low-cost treatment of mine water. Due to the relatively complex treatment process and high treatment costs, the treatment of highly mineralized mine water is challenging and a hotspot in current mine water treatment research [30].

Coal-based solid waste filling mining technology conforms to the development needs of “carbon peak and carbon neutrality”, and it is conducive to promoting high-quality coal mining, reducing environmental damage, and developing green low-carbon processes. In recent years, filling projects have been rapidly and widely carried out in Northwest China, especially in the Yushenfu mining area. For example, filling mining projects have been carried out in 13 mines (with an annual production capacity of nearly 200 million tons) belonging to the Shendong Company of the National Energy Group [31]. However, at present, there are still very limited studies on the mechanical characteristics of the filling concrete prepared with the widely-used highly mineralized mine water [5,32], and no systematic research has been performed on the performance of filling concrete, resulting in a lack of information for the design of fillings in regards to control of surface subsidence, groundwater loss, gas emission, land occupation damage, and mine pressure disasters (such as rock bursts).

Herein, mine water from the Shanghai Miao mining area in Ordos City was selected for the research studies [9], and we prepared filling concrete with highly mineralized mine water. We focused our investigation on the hardening characteristics of the filling concrete prepared with different dosages of highly mineralized mine water at different ages.

## 2. Materials and Methods

### 2.1. Materials

**Figure 1:**
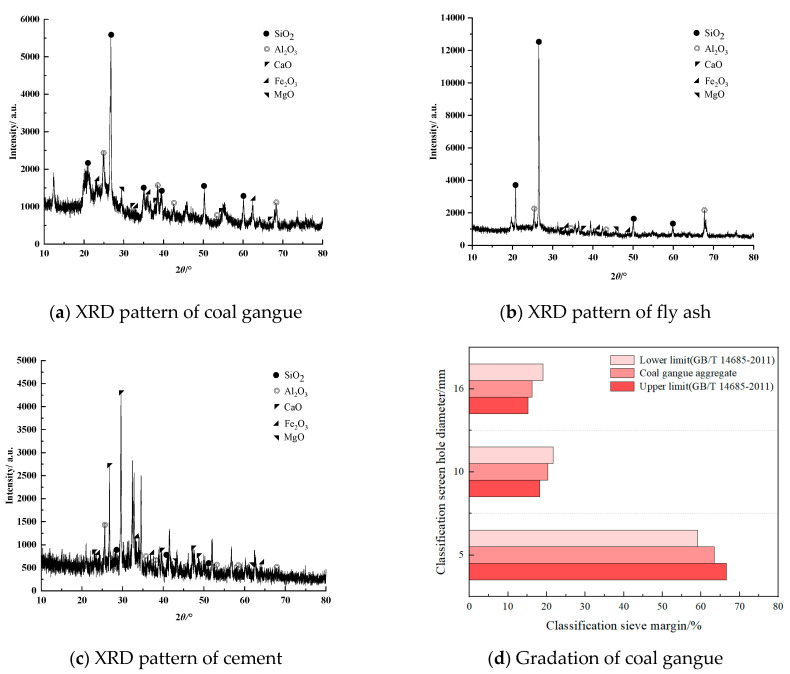
XRD patterns of raw materials and particle size distribution of coal gangue.

The main materials used in the test were P.O 42.5 ordinary Portland cement, fly ash, and coal gangue. The main chemical parameters of these raw materials are shown in Table 1. The cement was produced by China Resources Cement Co., Ltd. (Tai’an, China). The mine water, Grade-Ⅱ fly ash, and coal gangue all came from the Shanghai Miao mining area in Ordos. The maximum particle size of the coal gangue was 16 mm, and the particle size distribution is shown in Figure 1d. The chemical composition of the mine water is shown in Table 2, and the water used in the control experiment was domestic tap water.

According to the water quality analysis results in Table 2, the ion concentrations of Na^+^, Ca^2+^, Cl^−^, and SO_4_^2−^ are high, and the water hardness is high. The pH is 7.20, indicating the water is close to neutral, the salinity is over 1000 mg/L, and the mine water is classified as having a high mineralization degree [16].

X-ray diffraction (XRD) was used to analyze the composition of the selected test materials, and the results are shown in Figure 1a–c. The dominant chemical components of coal gangue, fly ash, and cement are SiO_2_, SiO_2_, and CaO, respectively, which are consistent with the results of the main chemical composition of each test material in Table 1.

### 2.2. Experimental Methods

**Figure 2:**
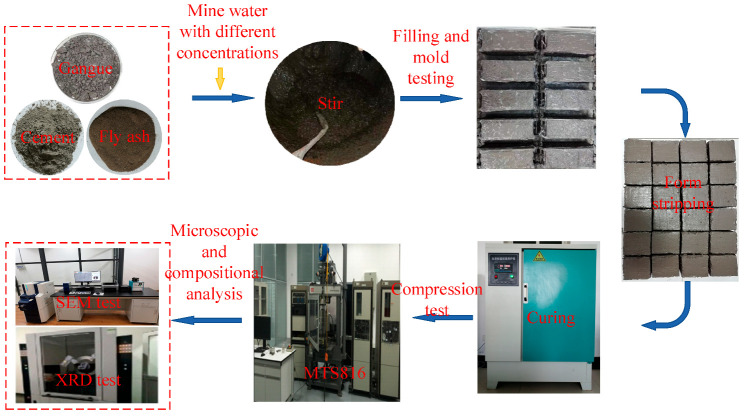
Flow chart of the experiment.

Figure 2 illustrates the flow chart of the experiment. The concrete was prepared according to five schemes with mine water dosages of 0, 25%, 50%, 75%, and 100%, respectively. The mass ratios of the raw materials were fly ash: cement: coal gangue = 1:1.5:8.3, and the solid mass concentration was 82%. For more specific details on the preparation, refer to the Method of Material Testing of Paste Filling in Mines (NB/T 51070-2017). The dimensions of specimens were 100 mm × 100 mm × 100 mm. Three specimens in each group of each scheme were tested for a total of 90 specimens. The schemes were numbered as T1 corresponding to a mine water dosage was 0, T2 (the mine water dosage was 25%), T3 (the mine water dosage was 50%), T4 (the mine water dosage was 75%), and T5 (the mine water dosage was 100%). The prepared filling concrete was placed in a curing box at a standard temperature of 20 °C and a relative humidity of 90% for curing. The curing ages were set as 1 d, 14 d, 28 d, 42 d, 56 d, and 70 d. The uniaxial compressive strength (σ_C_) values of the specimens in the first stage were tested using an MTS816 testing machine with a loading rate of 0.5 MPa/s. The data were recorded, and the average values of the three specimens in each group is presented as σ_C_ of this group. In the second stage measurements, powdered samples were prepared from the damaged specimens at 1 d, 28 d, and 70 d, respectively. The composition analysis was performed with XRD, and the microstructure of the damaged specimens was observed with SEM.

## 3. Long-Age Mechanical Characteristics of Filling Concrete

**Figure 3:**
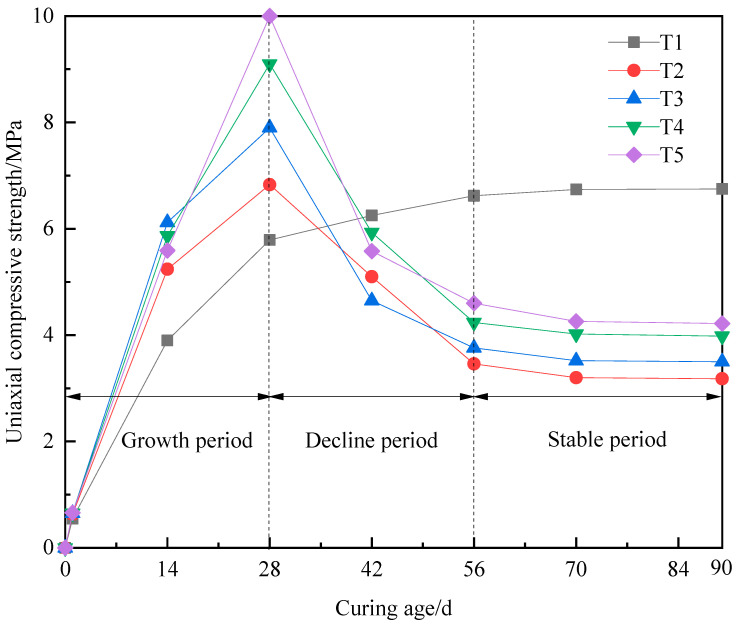
Diagram of compressive strength relationship of different samples with curing age.

Table 3 provides the results, and Figure 3 shows the changes in the compressive strength of the different filling concretes at different curing ages.

As can be seen from Figure 3, the trends in σ_C_ with curing time in T2, T3, T4, and T5 are basically identical. The curves can all be divided into three stages: a growth period, a decline period, and a stable period.

(1) The first stage, i.e., the growth period in σ_C_.

As can be seen in Figure 3, σ_C_ of T2, T3, T4, and T5 first shows rapid growth, and the growth rate is more significant than that seen for T1. After curing for 1 d, the σ_C_ values of T2, T3, T4, and T5 reached 0.63 MPa, 0.65 MPa, 0.65 MPa, and 0.66 MPa, respectively, and were similar to T1. After curing for 28 d, the σ_C_ values of T2, T3, T4, and T5 reached 6.83 MPa, 7.9 MPa, 9.1 MPa, and 10 MPa, respectively, which corresponded to increases of 18.0%, 36.4%, 57.2%, and 72.7%, respectively, compared to T1. The main reason for this increase is that the hydration reactions during the curing process can produce ettringite, calcium silicate gel, and a small amount of gypsum and Friedel’s salt. These products fill the internal pores and significantly increase the compactness. The reason why the σ_C_ value of the specimens with different dosages of mine water grows faster than those without mine water is that the chloride ions and sulfate ions in the highly mineralized mine water react with CaO·Al_2_O_3_(C_3_A) and ettringite to generate a new hydration product, i.e., Friedel’s salt, which fills the internal pores and makes the structure more compact.

(2) The second stage, i.e., the rapid decline period of σ_C_.

The changes in σ_C_ of T2, T3, T4, and T5 specimens are as follows. After curing for 28 d, σ_C_ decreases rapidly and reaches 3.46 MPa, 3.76 MPa, 4.24 MPa, and 4.6 MPa, respectively, at 56 d. In contrast, σ_C_ of T1 slowly increases until 70 d. The reason for the rapid decline is that more hydration products, i.e., ettringite and Friedel’s salt, are generated with increasing curing time which further expand the sample volume. As a result, micro-cracks begin to appear on the surface of concrete, and the cracks propagate and extend with the increase in curing age, resulting in a rapid decline of σ_C_.

(3) The third stage, i.e., the stable period of σ_C_.

After curing for 56 d, σ_C_ of T2, T3, T4 and T5 decreased slowly, mainly because the production of hydration products Friedel’s salt and ettringite in the specimen increased slowly, which showed that σ_C_ gradually tended to be stable. After curing for 90 days, σ_C_ is 3.18 MPa, 3.5 MPa, 3.98 MPa, and 4.22 MPa, respectively.

## 4. Formation Mechanism of the Long-Age Mechanical Characteristics of Filling Concrete

### 4.1. Microstructural Analysis of Filling Concrete Prepared without Highly Mineralized Mine Water (C_N-MW_)

**Figure 4:**
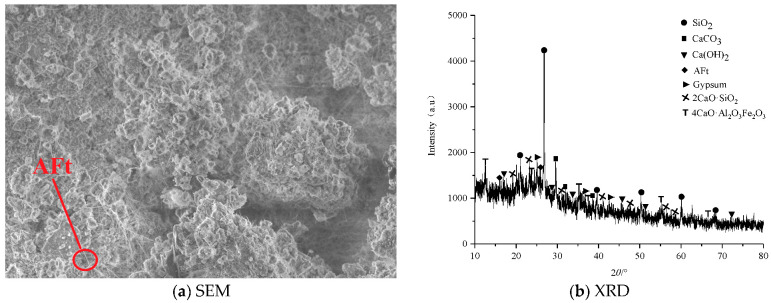
SEM image and XRD pattern of T1 specimen.

Figure 4 shows an SEM image of the morphology and XRD pattern of the filling concrete prepared without highly mineralized mine water (T1). As shown in Figure 4a, there are some amorphous clusters of different sizes inside the specimen and a small number of needle-rod crystals. Figure 4b shows that there are diffraction peaks corresponding to SiO_2_ around 25° and 50°, diffraction peaks corresponding to calcium carbonate around 30° and 40°, diffraction peaks corresponding to gypsum around 25°, 35°, and 40°. There are also high intensity diffraction peaks corresponding to calcium hydroxide at around 15° and 30° and low intensity diffraction peaks corresponding to ettringite at around 15° and 25°.

### 4.2. Microstructural Characterization for Filling Concrete with Different Dosages of Highly Mineralized Mine Water and the Formation Mechanism (C_MW_)

**Figure 5:**
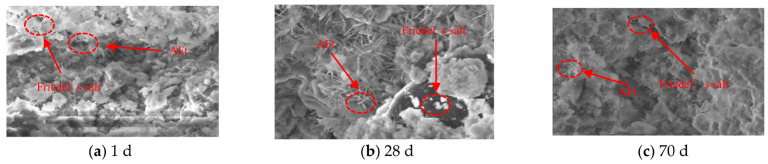
SEM images of T3 specimen at 1 d, 28 d, and 70 d.

**Figure 6:**
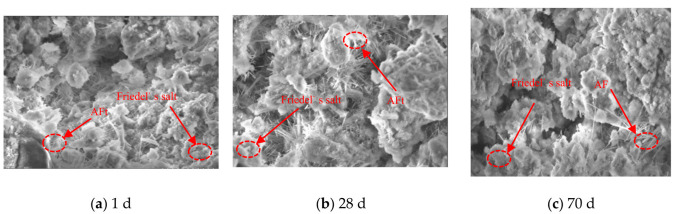
SEM images of T5 specimen at 1 d, 28 d, and 70 d.

By comparing Figure 5 with Figure 6, it can be seen that T5 specimen shows more obvious changes in microscopic products and pore structure compared with T3 specimen. After curing for 1 d, a small number of thin and short needle-rod crystals are formed as well as amorphous substances. After curing for 28 d, a large number of needle-rod crystals are evenly distributed in the samples, and the gaps between crystals decrease. After curing for 70 d, the needle-rod crystals are damaged and are disordered with shorter lengths and larger inter-crystal gaps.

**Figure 7:**
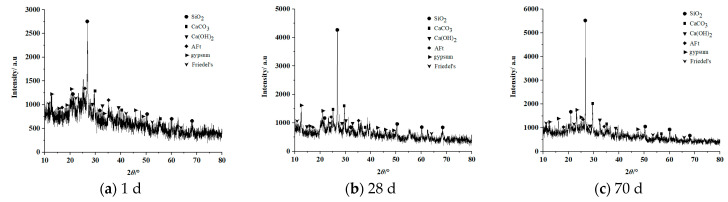
XRD patterns of T3 specimen at 1 d, 28 d, and 70 d.

**Figure 8:**
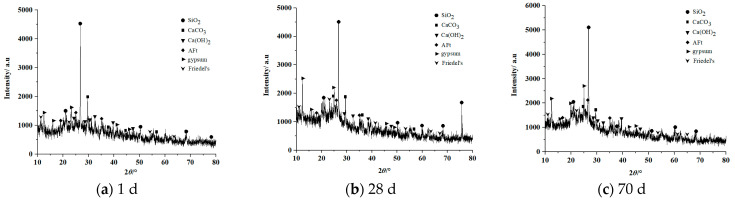
XRD patterns of T5 specimen at 1 d, 28 d, and 70 d.

Figure 7 and Figure 8 show the XRD patterns of the filling concrete prepared with highly mineralized mine water dosages of 50% (T3) and 100% (T5) after different times.

Figure 7 indicates that after curing for 1 d, the dominant hydration product in T3 is ettringite (3CaO·Al_2_O_3_·3CaSO_4_·32H_2_O, which is denoted as AFt for short). The corresponding diffraction peaks can be observed within the diffraction angle range of 20~40°. In addition, Friedel’s salt and gypsum are also generated. The diffraction peaks corresponding to Friedel’s salt can be seen at 10°, 22°, 55°, and 60°, and those of gypsum at angles of 12°, 15°, and 45°. The diffraction peak corresponding to calcium hydroxide appears near 25° and is lower in intensity than the peak seen in the data for T1. After curing for 28 d, the main hydration products are ettringite and Friedel’s salt. Compared to the XRD pattern at 1d, the intensity of the ettringite diffraction peak at 25° is higher, the intensity of the Friedel’s salt peak 22° is higher, and the intensity of the calcium hydroxide peak at a diffraction angle of 15° is significantly reduced due to the formation of Friedel’s salt, which consumes some calcium hydroxide inside the specimen. After curing for 70 d, the amount of the main hydration products, namely ettringite and Friedel’s salt, increases significantly. The peak intensity near a diffraction angle of 35° corresponding to ettringite is enhanced significantly, and the intensity of the peak from Friedel’s salt near a diffraction angles of 10° and 22° is also enhanced significantly.

Compared with the T3 specimen in Figure 7, the T5 specimen in Figure 8 has similar phase compositions at the different cure ages, mainly Friedel’s salt, ettringite, silica, calcium carbonate, calcium hydroxide, and gypsum. Notably, the intensities of the diffraction peaks of these six phases are higher overall than those for T3 specimen. This indicates that the amount of hydration products in the concrete prepared with 100% highly mineralized mine water is higher than in the sample prepared with a highly mineralized mine water dosage of 50%.

### 4.3. Mechanism Determination

The filling materials mainly react with Cl^−^ and SO_4_^2−^ in the highly mineralized mine water. During curing for 0~28 d, the strength of the concrete gradually increases, and a variety of minerals in the cement can react with water to induce hydration reactions. The hydration products are hydrated calcium silicate, calcium hydroxide, and hydrated tricalcium aluminate, and the reaction formulas are as follows:

2(2CaO·SiO_2_) + 4H_2_O→3CaO·2SiO_2_·3H_2_O + Ca(OH)_2_ (1)

3CaO·Al_2_O_3_ + 6H_2_O→3CaO·Al_2_O_3_·6H_2_O (2)

4CaO·Al_2_O_3_·Fe_2_O_3_ + 7H_2_O→3CaO·Al_2_O_3_·6H_2_O + CaO·Fe_2_O_3_·H_2_O (3)

Free Ca^2+^ can react with free SO_4_^2−^ to produce gypsum following reaction Formula (4). Meanwhile, SO_4_^2−^ can also react with Ca(OH)_2_, CaO, and Al_2_O_3_ to produce ettringite and gypsum dihydrate as hydration products as shown in reaction Formulas (5) and (6):

Ca^2+^ + SO_4_^−^ = CaSO_4_ (4)

3CaO·Al_2_O_3_·6H_2_O + 3Ca(OH)_2_ +3SO_4_^2−^ + 26H_2_O→3CaO·Al_2_O_3_·3CaSO_4_·32H_2_O + 6OH^−^ (5)

CaSO_4_ + 2H_2_O = CaSO_4_·2H_2_O (6)

Cl- can combine with CaO·Al_2_O_3_(C_3_A) that does not participate in hydration reaction to generate Friedel’s salt directly. On the other hand, Cl^−^ can replace SO_4_^2−^ in the formation of ettringite to generate Friedel’s salt. The reaction formulas are as follows:

Ca(OH)_2_ + 2Cl^−^ = CaCl_2_ + 2OH^−^ (7)

C_3_A + CaCl_2_ + 10H_2_O = C_3_A·CaCl_2_·10H_2_O (8)

C_3_A·3CaSO4·32H_2_O + 2Cl^−^ = C_3_A·CaCl_2_·10H_2_O + 2(CaSO_4_·2H_2_O) + SO_4_^2−^ + 18H_2_O (9)

As the curing age increases, the dominant hydration products, i.e., hydrated calcium silicate gel and hydrated tricalcium aluminate gel, strongly bond to the ettringite. Friedel’s salt is an insoluble and expansive hydration product that can fill the gaps inside the filling materials, thus making the structure of the specimen more compact. As a result, the strength of the specimen increases rapidly during curing from 0 to 28 d. With a further increase in curing age from 28 to 56 d, excessive Friedel’s salt and ettringite are generated inside the filling concrete. Due to the high expansibility of both products, expansion stresses are generated inside the filling paste. The expansion stress gradually increases with curing time which results in crack formation. As the paste becomes less compact, the two products change from their original excitation state to a final erosion state, which further aggravates the cracking process and eventually leads to the observed decrease in the strength of the specimens. From 56 to 70 d, the amount of hydration products, i.e., Friedel’s salt and ettringite, increases slowly following Reaction (5) and (9). As a result, the compressive strength of the filling concrete specimens increases slowly and tends to stabilize at long times.

## 5. Results and Discussion

At present, much research literature on filling materials mainly focuses on aggregate and cementing materials, and there are few studies on mixed water. However, in actual production, coal mining will inevitably discharge a large amount of mine water, and most coal mines are located in water-scarce areas. Therefore, the study of mine water as mixing water is conducive to alleviating the current situation of coal mine water shortage, improving the level of green mine construction and clean production, and promoting the development of ecological civilization in mining areas.

This paper provides an overview of the primary traits of mine drainage, which include high levels of suspended solids, salt, and acidity. Additionally, the study discusses the current state of mine water treatment technologies and associated costs. Notably, highly mineralized mine water has become a major challenge and research area in mine water treatment. To address this issue, a proposed solution is the “waste liquid utilization” approach, where mine water with high salinity is repurposed as a filling material for return filling.

In summary, we proposed a test method for the mechanical properties of long-aged filling concrete prepared with highly mineralized mine water and revealed that there are three-stages in the hardening characteristics, rather than the two seen in conventional concretes. We also pointed out an improvement direction for the material gradation.

(1) The mechanical properties and mechanism of long-aged C_MW_ containing with different dosages of highly mineralized mine water were studied, and C_MW_ showed three-stage hardening behavior. The trends in σ_C_ of the filling concrete specimens with highly mineralized mine water dosages of 25%, 50%, 75%, and 100% changed after 28 days and began to decrease with increasing curing age. At 0~28 d, σ_C_ increased with an increase in the mine water dosage; at 28~56 d, σ_C_ decreased with an increase in the mine water dosage; and at 56~70 d, σ_C_ was comparable for the different mine water dosages. At the curing age of 70 d, the σ_C_ values of C_MW_ with mine water dosages of 25%, 50%, 75%, and 100% were only 0.47, 0.52, 0.60, and 0.63 times those of C_N-MW_, respectively.
(2) Analysis of the microscopic structure showed that, compared with the C_N-MW_ specimen, diffraction peaks corresponding to Friedel’s salt appeared in the XRD patterns of C_MW_ with mine water dosages of 25%, 50%, 75%, and 100%, and that the intensities of diffraction peaks increased with an extension of the curing age, where the longer the curing age, the higher the Friedel’s salt content. The main hydration products in C_MW_ were Friedel’s salt and ettringite. The amounts of hydration products generated in C_MW_ prepared with a mine water dosage of 100% were higher overall than in C_MW_ prepared with a mine water dosage of 50%. As a result, the compressive strength σ_C_ of the C_MW_ specimen containing 100% mine water was higher than that of C_MW_ specimen with a 50% mine water dosage.

However, it should be noted that the reduction of strength after 28 d will affect the filling effect. Therefore, more studies on improving the material ratio will be subsequently carried out to improve the hardening strength of long-aged highly mineralized mine water-filled concrete after 28 days in order to reduce strength loss. Moreover, it is advisable to undertake a more comprehensive literature review study concurrently. In addition, there are other challenges that need to be tackled in the future. For example, the current experimental study only accounts for a limited number of factors that influence mine water. It is worth noting that the mixing water utilized in mining areas contains not only SO_4_^2−^ and Cl^−^, but also a substantial quantity of Fe^2+^, Ca^2+^, and K^+^ plasma. As a result, various ionic solutions must be prepared to correspond with different types of mixing water. In the present work, the impact of mixing water with intricate mineralized content on the performance of filling concrete was studied.

## Figures and Tables

**Table 1:** Main components of the experimental raw materials.

Raw Materials	SiO_2_/%	Al_2_O_3_/%	Fe_2_O_3_/%	CaO/%	MgO/%
Coal gangue	32.6	27.3	14.2	15.9	2.10
Fly ash	51.50	30.6	6.00	3.72	0.93
P.O 42.5	7.5	24.18	19.10	39.20	3.02

**Table 2:** Main components of mine water.

Ion Species	Content	
	Mass Concentration/mg·L^−1^	Molar Concentration/mmol·L^−1^
Na^+^	1513.00	65.82
Ca^2+^	40.42	2.02
Mg^2+^	127.19	10.46
CO_3_^2−^	28.52	0.95
HCO^−^	568.84	9.32
SO_4_^2−^	883.29	18.39
Cl^−^	1760.99	49.66

**Table 3:** Compressive strength of the filling concrete at different curing ages.

Curing Age /d	Paste Strength/MPa				
	T1	T2	T3	T4	T5
1	0.55	0.63	0.65	0.65	0.66
14	3.9	5.24	6.12	5.87	5.59
28	5.79	6.83	7.9	9.1	10
42	6.25	5.1	4.65	5.93	5.58
56	6.62	3.46	3.76	4.24	4.6
70	6.74	3.2	3.52	4.02	4.26
90	6.75	3.18	3.5	3.98	4.22
